# Long Non-Coding RNAs in Multiple Myeloma

**DOI:** 10.3390/genes9020069

**Published:** 2018-02-01

**Authors:** Lucia Nobili, Domenica Ronchetti, Luca Agnelli, Elisa Taiana, Cristina Vinci, Antonino Neri

**Affiliations:** Department of Oncology and Hemato-Oncology, University of Milan, and Hematology, Fondazione Ca’ Granda IRCCS Policlinico, 20122 Milan, Italy; lucia.nobili@unim.it (L.N.); domenica.ronchetti@unimi.it (D.R.); luca.agnelli@unimi.it (L.A.); elisa.taiana@unimi.it (E.T.); cristina.vinci@unimi.it (C.V.)

**Keywords:** long non-coding RNAs (lncRNAs), multiple myeloma (MM), expression profiling, transcription regulation

## Abstract

Multiple myeloma (MM) is an incurable disease caused by the malignant proliferation of bone marrow plasma cells, whose pathogenesis remains largely unknown. Although a large fraction of the genome is actively transcribed, most of the transcripts do not serve as templates for proteins and are referred to as non-coding RNAs (ncRNAs), broadly divided into short and long transcripts on the basis of a 200-nucleotide threshold. Short ncRNAs, especially microRNAs, have crucial roles in virtually all types of cancer, including MM, and have gained importance in cancer diagnosis and prognosis, predicting the response to therapy and, notably, as innovative therapeutic targets. Long ncRNAs (lncRNAs) are a very heterogeneous group, involved in many physiological cellular and genomic processes as well as in carcinogenesis, cancer metastasis, and invasion. LncRNAs are aberrantly expressed in various types of cancers, including hematological malignancies, showing either oncogenic or tumor suppressive functions. However, the mechanisms of the related disease-causing events are not yet revealed in most cases. Besides emerging as key players in cancer initiation and progression, lncRNAs own many interesting features as biomarkers with diagnostic and prognostic importance and, possibly, for their utility in therapeutic terms as druggable molecules. This review focuses on the role of lncRNAs in the pathogenesis of MM and summarizes the recent literature.

## 1. Introduction

Multiple myeloma (MM) is a fatal malignant proliferation of antibody-secreting bone marrow (BM) plasma cells (PCs) that accounts for 10% of all hematological malignancies with an incidence in western countries of about 3–5 per 100,000. MM is characterized by an extensive clinical variety ranging from the presumed premalignant condition, called monoclonal gammopathy of undetermined significance (MGUS), to extramedullary myeloma and plasma cell leukemia (PCL) [1,2,3]. Despite the remarkable improvements in the treatment and patient care [4], MM remains an incurable disease.

MM shows a high genomic time- and phase-dependent instability and consequently a very complex karyotype that leads this plasma cell dyscrasia to more resemble solid than haematologic tumors [1,5,6]. Nearly half of MM tumors are hyperdiploid, associated with nonrandom trisomies of odd chromosomes. The remaining tumors are non-hyperdiploid and frequently associated with the constitutive activation of *CCND1* (11q13), *CCND3* (6p21), *MAF* (16q23), *MAFB* (20q11), or *FGFR3/MMSET* (4p16.3) genes consequent to chromosomal translocations involving the immunoglobulin heavy-chain locus (*IGH*) on chromosome 14q32. The mechanisms underlying this dichotomic pattern have not been elucidated yet. Hyperdiploid patients have a generally better prognosis, whereas the t(4;14) and t(14;16) translocations are associated with a poor prognosis, either under conventional or high-dose therapy. In addition, allelic imbalances of specific genomic regions, including 13q, 17p13, 1p, 16q, 14q losses and 1q gains, are adversely linked to prognosis [5,6]. The high-risk chromosomal aberrations del (17p13), t(4;14), and 1q21 have been identified as adverse prognostic factors also in asymptomatic smoldering MM (SMM), independent of the tumor burden, whereas hyperdiploidy, contrary to active myeloma, seems to be an adverse prognostic factor in SMM [7]. Overall, transcriptional data have provided valuable information on the genes aberrantly expressed in MM, further supporting their high biological heterogeneity and showing their promising prognostic role [8].

During the last two decades of research, thanks to the impressive development and application of high-throughput sequencing techniques, it has been demonstrated that, although a large fraction of the human genome is actively transcribed [9,10], non-coding RNAs (ncRNAs), namely the RNA fraction not translated into canonical functional proteins, account for the majority of transcripts. Increasing evidence suggests their huge impact on several molecular mechanisms: in particular, the roles of ncRNAs in supporting cellular homeostasis and gene expression regulation and in governing different pathologies are recently emerging.

According to their size, ncRNAs have been arbitrarily categorized into short and long ncRNAs (lncRNAs), the latter being longer than 200 nucleotides. Short ncRNAs can be further subdivided into various categories, including microRNAs (miRNAs), small interfering RNAs, PIWI-associated RNAs and small nucleolar RNAs. Dysregulated short ncRNAs, especially miRNAs, are known to have important functions in virtually all types of cancer, including MM. Further, miRNA profiling has assumed a crucial value in tumor diagnosis and prognosis and in predicting the response to therapy [11]. Notably, miRNAs have also gained importance as innovative therapeutic targets in cancer, including MM [12,13,14].

LncRNAs are a heterogeneous group representing more than half of the mammalian non-coding transcriptome. They participate in various biological processes, such as transcriptional gene regulation, maintenance of genomic integrity, X-chromosome inactivation, genomic imprinting, cell differentiation, and development. LncRNAs are transcribed from introns, exons, intergenic or intra-genic regions, promoter regions, 3′- and 5′-UTRs; therefore, they may represent intronic, intergenic, bidirectional, and antisense- or sense-overlapping sequences [15,16]. The catalog of known human lncRNA genes and transcripts has been growing impressively over the last few years and is still evolving. The largest repositories currently contain more than 60,000 human annotated lncRNA genes (e.g., 65,694 LNCpedia v4.1; 96,308 Noncode v5.0), with many loci generating multiple transcripts. Such a discrepancy is mainly attributable to the wide or stringent criteria used to include sequences based on RNA-sequencing studies predictions [17,18].

Not greatly different from mRNAs as it regards structure and biogenesis, lncRNAs can be polyadenylated and may operate in either nuclear or cytoplasmic fractions. Their expression is developmentally regulated and it is restricted to specific cell or tissue types to a greater extent than mRNA [15]. The adaptability due to their secondary structure allows lncRNAs to form binding sites for the interaction with proteins, DNA, and other RNA molecules. Thanks to specific substructures, lncRNAs can then function as guides, tethers, decoys, and scaffolds. LncRNAs can regulate gene expression at three levels, specifically, the transcriptional, post-transcriptional, and chromatin modification levels [16,19,20,21,22,23,24]. Although the mechanisms underlying the function of most lncRNAs are not fully understood, the deregulation of distinct lncRNAs has been reported to promote tumor formation, progression, and metastasis in many types of cancer, including hematologic malignancies [20,21]. Interestingly, because of their presence in body fluids, including urine and blood, although at a low level of expression, circulating cell-free lncRNAs are promising putative non-invasive diagnostic and prognostic biomarkers in cancer patients [25].

Even though it is still in its beginnings, the knowledge of the role of lncRNAs in MM is progressively expanding. In this review, we provide a compendium of the lncRNAs reported to be deregulated in MM and, therefore, putatively implicated in its pathogenesis and clinical outcome.

## 2. LncRNAs in Multiple Myeloma

### 2.1. MALAT1

The expression of *MALAT1* (metastasis-associated lung adenocarcinoma transcript 1), a putative oncogenic lncRNA transcribed from chromosome 11q13 and overexpressed in several solid tumors [26,27], was firstly investigated by Cho et al. [28] in BM mononuclear cells from MM patients with different disease status at diagnosis, showing its significant overexpression compared to normal controls. Moreover, patients who relapsed or had disease progression showed a significant increased expression of *MALAT1*. These findings were further supported by Ronchetti et al. investigating, by expression microarray, a large cohort of patients encompassing all the major different forms of PC dyscrasias: notably, an association was found between *MALAT1* upregulation and molecular pathways involved in cell cycle regulation, p53-mediated DNA damage response, and mRNA maturation processes [29]. More recently, other groups found high expression levels of *MALAT1* in BM mononuclear cells from untreated MM patients with different disease status, as well as in MM cell lines, along with a significant reduction of its expression in complete remission patients [30,31,32]. In the study from Gao et al. [30], the expression trend of *MALAT1* strictly resembled that of the DNA-binding protein high mobility group box 1 (HMGB1), which acts as a mediator of autophagy in the cytoplasm. HMGB1 was previously found overexpressed in MM and shown to sustain the survival and proliferation of myeloma cells [33]. *MALAT1* knockdown in MM cell lines and in a mouse model [30] reduced HMGB1 expression levels as well as the expression of Beclin-1 and LC3B, both related to autophagy. The direct binding between *MALAT-1* and HMGB1 might be partially responsible for an increased ubiquitination and degradation of the protein. *MALAT1* knockdown promoted also a significant reduction of viability and an increase of apoptosis in MM cells, whereas the combined *HMGB1* overexpression was able to abolish all the mentioned effects. These findings suggest that *MALAT1* favors autophagy in MM by upregulating *HMGB1* expression, thus supporting its role in the suppression of apoptosis and the promotion of tumor cell survival. The induction of apoptosis in *MALAT1*-silenced cells might involve a down-regulation of cyclin D1 and cyclin E, the activation of caspase-3 and caspase-9, the increased level of the pro-apoptotic protein BAX, and the decreased level of the anti-apoptotic protein BCL-2 [31]. Worth noting is also that *MALAT1* has been recently linked with the extramedullary dissemination of MM, often developing after several chemotherapeutic interventions and associated with a worse prognosis: not only a substantially greater expression of *MALAT1* was found in extramedullary MM, but even higher expression levels were shown in extramedullary PCs matched with the intramedullary ones from the same patients [32]. These findings extend previous data [29], thus suggesting a role for *MALAT1* in extramedullary dissemination. This hypothesis was further supported by the correlation of higher *MALAT1* expression with worse overall and progression-free survival (OS and PFS); in particular, *MALAT1* expression showed an independent influence on PFS, suggesting a possible role in drug resistance [32]. Furthermore, the positive correlation between the expression levels of *MALAT1* and heat shock protein 90 s (known to be induced in response to diverse cellular stress), together with the in vitro demonstration of *MALAT1* upregulation after treatment with proteasome inhibitors and doxorubicin, suggested that *MALAT1* overexpression might depend on, or at least be related to, the chemotherapeutic stress response [32].

Finally, *MALAT1* was recently shown to be overexpressed in mesenchymal stromal cells (MSCs) from MM patients, which led to the transcriptional activation of the neighboring antisense protein-coding gene *LTBP3* (latent TGF-β-binding protein) [34]. *LTBP3* is acknowledged as a positive regulator of the activity of TGF-β, which may get involved in the inhibition of terminal osteoblastogenesis in MM [35]. Actually, it was demonstrated that MALAT1 recruits the transcription factor SP1 on the *LTBP3* promoter, thus contributing to the increase of *LTBP3* expression. Conversely, a significant reduction of *LTBP3* transcription followed *MALAT1* knockdown [34].

### 2.2. MEG3

*MEG3* (maternally expressed gene 3) lncRNA, located at 14q32.2, is thought to act as a tumor suppressor through both p53-dependent and p53-independent mechanisms and to be controlled epigenetically at the expression level [36,37]. Studying the expression of *MEG3* in MM, Benetatos et al. observed hypermethylation of the differentially methylated region (DMR) of the *MEG3* promoter in about 60% of patients, which correlated with both disease stage and subtype [38]. Two-third of the patients with IgG MM and all of those with IgM exhibited the epigenetic alteration, whereas none of the patients with IgA had hypermethylated DMR. These findings led to suggest a possible involvement of *MEG3* lncRNA promoter hypermethylation and, consequently, of its reduced expression in MM tumorigenesis. Of interest, MSCs from MM patients (MM-MSCs) expressed *MEG3* to a lesser degree than those from normal donors (ND-MSCs) throughout osteogenic differentiation [39]. Moreover, the overexpression of *MEG3* was able to stimulate MM-MSCs to differentiate, while *MEG3* knockdown had a negative effect on the osteogenesis of ND-MSCs. On the basis of experimental investigations, *MEG3* promoted osteogenesis by targeting the transcription of *BMP4* (bone morphogenetic protein 4), a gene member of the *TGF* family. Both *MEG3* and *BMP4* map to chromosome 14, albeit with divergent transcription orientation. *MEG3* is supposed to interact directly with the transcription factor SOX2, triggering its separation from the *BMP4* promoter and then determining an increase of *BMP4* expression [39].

### 2.3. CRNDE

*CRNDE* (colorectal neoplasia differentially expressed), a lncRNA mapped at 16q12.2, was firstly recognized as upregulated in colorectal cancer [40]. Subsequently, the elevated expression of this lncRNA was found in the early stages of human development, in several solid tumors, as well as in leukemias and MM [41]. In their recent study, Meng et al. [42] found increased expression of *CRNDE* in MM cell lines and patients, associated with poor OS. *CRNDE* knockdown in U266 and RPMI-8226 cell lines inhibited proliferation and colony formation and led to increased apoptosis and cell cycle arrest in G0/G1 phase. The knockdown of *CRNDE* in U266 cells was associated with a considerable increment of miR-451 expression, otherwise downregulated in MM patients compared with normal controls. By means of bioinformatics analysis and dual-luciferase reporter assay, a putative complementary interaction between miR-451 and the 3′-UTR of *CRNDE* was shown. Furthermore, Meng et al. found a negative correlation of *CRNDE* with miR-451 in MM patients, suggesting that *CRNDE* might act in MM pathogenesis by negatively targeting miR-451. This hypothesis was reinforced by the observation that miR-451 inhibition was able to rescue the above described effects of *CRNDE* knockdown in U266 cell line, thus restoring the tumorigenesis of the cells [42].

### 2.4. UCA1

A possible prognostic impact of the expression level of lncRNA *UCA1* (urothelial cancer associated 1) in MM was recently postulated by Sedlarikova et al. [43] in a study analyzing the expression of 83 candidate lncRNAs in BM PCs of newly diagnosed MM patients compared to normal BM PCs of healthy donors (HD). Specifically, of the 27 lncRNAs identified as differentially expressed in MM patients, *UCA1* resulted significantly downregulated in MM samples compared to HD, also showing a negative and a positive correlation with albumin and monoclonal-Ig serum levels, respectively. Moreover, in MM patients higher levels of *UCA1* correlated with poorer OS and the occurrence of 1q21 gain or t(4;14) translocation, known unfavorable prognostic features of MM.

*UCA1*, mapped at 19p13.12, was firstly identified as greatly expressed in bladder transitional cell carcinoma [44]. A link between *UCA1* and the transcription factor CREB (cAMP response element-binding protein) was found in bladder cancer cells [45] indicating that *UCA1* might influence cell cycle regulation by activating CREB via PI3K–AKT pathway.

### 2.5. OIP5-AS1

Yang et al. discovered an inverse correlation between the high levels of miR-410 expression in newly diagnosed MM and lncRNA *OIP5-AS1* (*OIP5* antisense RNA 1), mapped on chromosome 15q15.1 [46]. Experimental evidences indicated that *OIP5-AS1* was able to inversely modulate the expression of miR-410 and its pro-oncogenic effects on cell proliferation, cell cycle progression, and apoptosis in NCI-H929 and RPMI-8226 cell lines. On the basis of the identification of *KLF10* (Kruppel Like Factor 10), involved in PTEN–AKT signaling activation, as a direct downstream target of miR-410 in MM, it was further demonstrated the contribution of *OIP5-AS1* in the KLF10–PTEN–AKT signaling pathway in MM cells, very likely through a negative regulation of miR-410. Overall, the loss of lncRNA *OIP5-AS1* induced miR-410 accumulation in MM cells leading to cell proliferation, cell cycle progression, and apoptosis inhibition via the KLF10–PTEN–AKT signaling axis.

## 3. lncRNA Expression Profiles in MM

Studies investigating the global lncRNA expression in MM are still limited and based on microarray data. In particular, there are two different studies concerning the same publicly available microarray datasets including a large group of 559 MM patients, profiled onto GeneChip^®^ 3′-in vitro transcription arrays querying the poly-A+ fraction of transcripts [47,48]. In the first investigation, Zhou et al. [47] examined a list of 2330 lncRNA by re-annotating such datasets and identified a four-lncRNA prognostic signature able to predict survival in MM patients. In particular, two lncRNAs, *RP1-43E13.2* and *RP4-803J11.2*, are located on chromosome 1p and 1q, respectively, frequently lost or gained in MM and related with poor prognosis [6,49,50,51]. The second study, based on custom lncRNA-oriented annotation files of the same datasets, identified 176 lncRNAs able to clusterize patients into two groups with significant differences in terms of OS [48] and predict the patient’s prognosis independently from β2-microglobulin, albumin, and LDH serum levels.

**Figure 1:**
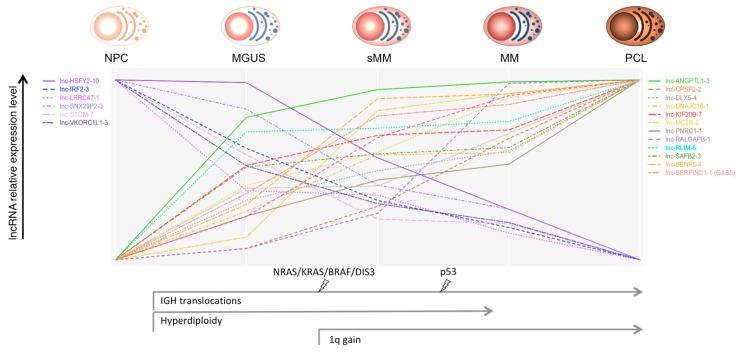
Oncogenic events, such as the main chromosomal aberrations and gene mutations, through the different stages of plasma cell (PC) dyscrasias are represented. The relative expression level (scaled to common y-axis range on the upper and lower values) of the long ncRNAs (lncRNAs) deregulated through the progressive stages of PC dyscrasias is shown [26]. Abbreviations: NPC = normal plasma cell; MGUS = monoclonal gammopathy of undetermined significance; sMM = smoldering multiple myeloma; MM = multiple myeloma; PCL = plasma cell leukemia.

Ronchetti et al. [29], by employing new-generation whole-transcript arrays, analyzed the global lncRNA expression profiles in a group of 259 patients, representative of all the main forms of PC dyscrasias, which included MGUS, asymptomatic SMM, truly overt and symptomatic MM, extra-medullary MM/PCL patients, together with nine HD. Among the 1852 lncRNAs queried by unique probes on the array, 230 were capable to discriminate normal and pathological samples and the different forms of PC dyscrasias. A group of 31 lncRNAs, including *MALAT1*, was specifically dysregulated in pathological specimens in comparison to normal BM PCs. In addition, the expression of 21 lncRNAs showed a progressive deregulation linked to the aggressiveness of the disease (Figure 1). In particular, *lnc-SENP5-4*, *lnc-CPSF2-2*, and *lnc-LRRC47-1* displayed a significantly different expression when a group of 19 MMs at diagnosis was matched with the corresponding relapse–PCL phases. Interestingly, the downregulation of *lnc-LRRC47-1* from normal PCs to MGUS and symptomatic myeloma was reported also by others [52]. The panel of 21 lncRNA included *GAS5* (growth arrest-specific 5)*,* mapped at 1q25, a tumor suppressor-like associated with cell cycle arrest and apoptosis [53,54,55,56,57]. Interestingly, *GAS5* was also significantly upregulated in MMs showing 1q gain, together with other six specific lncRNAs. Patients with del13 had a significant downregulation of *DLEU2* (deleted in leukemia 2), mapping at 13q14.3 and hosting the cell cycle inhibitory miRNAs 15a and 16-1 [58]. A significant correlation of *DLEU2* with miR-15a and miR-16-1 expression was shown. These findings support a gene-dose effect as a probable mechanism conditioning lncRNAs deregulation.

Finally, Ronchetti et al. expanded their investigation on the function of lncRNA dysregulation in PC dyscrasias by analyzing the association miRNA–lncRNA in MM, PCL, and normal PCs samples. The identification of lncRNA–miRNA pairs (*lnc-MCL1-2* and mir-17 gene family; *lnc-AGBL1-4* and mir-185-5p; *lnc-DLEU2* and miR-3175; *LINC00173* and miR-221) with a possible important interplay for MM biology, suggested an influence of miRNA–lncRNA communication on MM pathology [59].

A selection of lncRNAs deregulated in MM with potential relevance in the pathology is reported in Table 1.

## 4. Conclusions

LncRNAs are a large and composite family of transcripts that do not encode proteins, with an emergent crucial role in a variety of processes accompanying the biology of normal and cancer cells. Their tissue-specific expression and their easy detectability in body fluids confer them strong potential as biomarkers in the diagnosis and prognosis of many cancer types. The investigation of lncRNAs in MM is still limited, and more extensive profiling studies based on novel approaches, such as RNA sequencing, are needed. In addition, among the dysregulated lncRNAs in MM identified so far, only a few of them have been, at least in part, characterized. However, the available data suggest central roles for lncRNAs in different stages of PC dyscrasias and prompt to explore their relevance as prognostic biomarkers. Furthermore, the targeting of specific lncRNAs in MM have been scarcely investigated until now, hence, a more thorough understanding of their functions and mechanisms in the disease biology is certainly necessary. Improving the knowledge of the types and roles of different lncRNAs in MM will clearly represent a significant step forward in the current comprehension of its pathobiology. Indeed, it would provide valuable information about key cancer-promoting pathways in MM and be useful for a better diagnostic and prognostic assessment. Furthermore, lncRNA may represent excellent candidates for new anticancer approaches, mostly because of their cancer- and tissue-specific expression that could confer major advantages over other therapeutic options. Their better understanding in MM, may provide an important contribution for the development of novel lncRNA-based therapies for the disease.

## Figures and Tables

**Table 1:** Overview of selected lncRNAs with a putative pathogenetic role in multiple myeloma (MM).

lncRNA(s)/Alias	Loc	Func ^a^	Molecular Mechanism	Ref(s).	Tissue	Pts (n) ^b^	Method(s)
DLEU2	13q14.3	TS	Host of miR-15a/16-1 cluster targeting *BCL2*.	[29]	bone marrow (BM) plasma-cells (PCs)	259 + 9 healthy donors	Gene expression profiling
				[58]	chronic lymphocytic leukemia (CLL)	7 CLL (and 1 CLL cell line)	RT-PCR
GAS5	1q25.1	TS	Required for the inhibitory effects of mTOR antagonists. Regulated by mTOR pathway.	[29]	BM PCs	259 + 9 healthy donors	Gene expression profiling
				[53]	embryo and adult mouse tissues; Friend leukemia cells	N/A	Northern blot analysis; in situ hybridization
				[57]	paired tumor and adjacent normal breast epithelial tssues	21 (and several mammalian cell lines)	Semiquantitative and qRT-PCR
				[54]	lymphocytes from diffuse large B-cell lymphoma	1	RT-PCR
				[55]	renal cell carcinoma (RCC)	12 (and 1 RCC cell line + 1 nonmalignant renal cell line)	qRT-PCR
				[56]	paired non-small cell lung cancer and adjacent normal lung tissue	72	qRT-PCR
MALAT1	11q13	O	SP1 recruitment to the promoter of *LTBP3* gene regulating the bioavailability of TGF-β.	[26]	Hela cells	N/A	qRT-PCR
				[27]	HepG2 and HeLa cell lines	N/A	RT-PCR, immunofluorescence
				[28]	BM mononuclear cells from MM patients	124 + 20 healthy donors	qRT-PCR
				[29]	BM PCs	259 + 9 healthy donors	Gene expression profiling
				[30]	BM mononuclear cells from MM patients	60 + 10 healthy donors (and 2 MM cell lines)	qRT-PCR
				[31]	MM cell lines	not applicable	RT-PCR
				[32]	BM and extramedullary PCs	162 (and 5 MM cell lines)	RT-PCR
				[34]	BM mesenchymal stromal cells from MM patients	25 + 5 healthy donors	qRT-PCR
MEG3	14q32.2	TS	Interaction with p53. Regulation of *P53* gene expression. Enhancement of the expression of *BMP4* gene in MM-MSCs.	[36]	Human colon carcinoma and osteosarcoma cell lines	N/A	RT_PCR
				[38]	BM and peripheral blood from MM patients	21 + 10 healthy donors	Methylation-specific PCR
				[39]	BM mesenchymal stromal cells from MM patients	6 + 3 healthy donors	qRT-PCR
CRNDE	16q12.2	O	Negative targeting of miR-451.	[42]	BM PCs	77 + 19 healthy donors (and 5 MM cell lines)	qRT-PCR
UCA1	19p13.12	O	Cell cycle regulation by activation of CREB via PI3K-AKT pathway.	[43]	BM PCs	84 + 22 healthy donors	qRT-PCR
				[45]	Human bladder cancer cell line BLZ-211		Gene expression profiling
OIP5-AS1	15q51.1	O	Molecular sponge modulating miR-410.	[46]	BM PCs	97 + 14 healthy donors (and 3 MM cell lines)	qRT-PCR
lnc-SENP5-4/NCBP2-AS2	3q29	U	Not described.	[29]	BM PCs	259 + 9 healthy donors	Gene expression profiling
lnc-CPSF2-2	14q32						
lnc-LRRC47-1/TP73-AS1	1p36						
lnc-ANGPTL1-3	1q25						
lnc-WHSC2-2	4p16.3						

Abbreviations: ^a^ U = Uncharacterized; O = Oncogene; TS = Tumor Suppressor; ^b^ Pts (n) = Patients (number). N/A: not applicable. BM = bone marrow; MSCs = mesenchymal stromal cells.
